# Association between triglyceride glucose-waist height ratio index and overactive bladder: based on NHANES 2005-2018

**DOI:** 10.3389/fendo.2025.1541218

**Published:** 2025-04-15

**Authors:** Haiyan Mao, Tong Lin, Shanshan Huang, Zhenye Xie, Zhikui Chen

**Affiliations:** ^1^ Department of Critical Care Medicine, Ningbo Medical Center Lihuili Hospital, Ningbo, China; ^2^ Cardiovascular Medicine, Ningbo Medical Center Lihuili Hospital, Ningbo, China

**Keywords:** triglyceride glucose-waist height ratio, overactive bladder, metabolic disease, retrospective study, NHANES

## Abstract

**Background:**

The triglyceride glucose-waist height ratio (TyG-WHtR) index is a useful marker for predicting the risk of cardiovascular and metabolic diseases. Metabolic diseases are known to be high-risk factors for overactive bladder (OAB). However, no studies have explored the association between the TyG-WHtR index and the risk of developing OAB.

**Methods:**

Data from the National Health and Nutrition Examination Survey (NHANES) was utilized, and a weighted multivariate logistic regression analysis was conducted to investigate the relationship between TyG-WHtR and OAB. Subgroup analyses and interaction tests were also performed. Additionally, sensitivity analyses were conducted to validate the robustness of the findings. A smooth curve fitting and threshold effect analysis explored the nonlinear relationship between TyG-WHtR and the risk of developing OAB. The predictive value of the TyG-WHtR index for OAB was assessed using Receiver Operating Characteristic (ROC) curves, and the area under the ROC curve (AUC) was calculated.

**Results:**

A total of 14,652 adults aged 20 and above were included in this study. After weighting, the population size was estimated to be 197,598,146.7, among which 37,872,284.55 individuals were diagnosed with OAB. The median TyG-WHtR for the entire population was 4.98, while it was 5.44 for those with OAB. Weighted logistic regression analysis revealed a significant positive association between TyG-WHtR and the occurrence of OAB (OR=1.646; 95% CI: 1.562, 1.735; *P*<0.001). This positive association remained significant even after adjusting for confounding factors (OR=1.310; 95% CI: 1.157, 1.484; *P*<0.001). Sensitivity analysis demonstrated the robustness of the results. Subgroup and interaction analyses indicated that the impact of the TyG-WHtR index on OAB might be influenced by gender (OR=1.323; 95% CI: 1.138, 1.538; *P*<0.001) and age (OR=1.426; 95% CI: 1.180, 1.724; *P*<0.001). Smooth curve fitting and threshold effect analysis revealed a threshold of 3.579. ROC curve analysis demonstrated that the TyG-WHtR index has a good predictive ability for OAB (AUC=0.647; 95% CI: 0.636, 0.657).

**Conclusions:**

The TyG-WHtR index is significantly positively associated with the occurrence of OAB and could potentially serve as a novel risk predictor for OAB. Future research is needed to validate findings, explore causality, and improve early detection through multifactorial models across diverse populations.

## Introduction

Overactive bladder (OAB) is a common urological condition characterized by an overactive voiding reflex in the absence of urinary tract infection or other identifiable pathological changes. It is marked by symptoms such as urgency urinary incontinence (UUI) and frequent nocturia (1). The global prevalence of OAB is estimated to be around 11-16%, affecting over 400 million people worldwide (2). OAB imposes a significant economic burden on patients, with the average monthly cost of urinary symptoms for American adults being approximately $3,003. The healthcare expenses for OAB patients are more than 2.5 times higher than those without OAB (3). Furthermore, OAB significantly impacts patients’ quality of life; frequent trips to the bathroom can lead to reduced social activity, hinder work and daily life, and cause embarrassment, anxiety, and even depression. Additionally, OAB patients often suffer from severe autonomic dysfunction, which is highly correlated with the incidence of cardiovascular diseases (4, 5).

The exact etiology and pathogenesis of OAB remain unclear (6). Neurological disorders such as stroke, metabolic diseases, bladder factors, and lifestyle choices are all considered high-risk factors for the development of OAB (7, 8). Current treatment strategies for OAB emphasize long-term comprehensive management, including lifestyle changes, regular pelvic exercises, pharmacotherapy (such as vaginal estrogen, anticholinergic drugs, and β3 agonists), and invasive treatments (like sacral nerve modulation, percutaneous tibial nerve stimulation, and surgery) (9–11). However, these treatments mainly alleviate symptoms and are generally unable to cure OAB.

Obesity and diabetes are recognized as significant risk factors for OAB (8). Both conditions are frequently associated with insulin resistance (IR) (12). The triglyceride-glucose (TyG) index, calculated from fasting triglyceride and glucose levels, is a reliable marker of insulin resistance (13). Compared to traditional methods for assessing insulin resistance, such as the hyperinsulinemic-euglycemic clamp or the homeostasis model assessment of insulin resistance (HOMA-IR), the TyG index is simpler, more convenient, and cost-effective (14). The TyG index is a surrogate marker for identifying individuals at risk of metabolic diseases (such as type 2 diabetes and non-alcoholic fatty liver disease) and cardiovascular diseases (15–17). The waist-to-height ratio (WHtR) is an indicator of obesity. It has been found to more accurately reflect abdominal obesity and predict the risk of cardiovascular and metabolic diseases compared to traditional body mass index (BMI) (18, 19). Studies have demonstrated that combining the TyG index with obesity metrics provides better IR and cardiovascular risk. Compared to other TyG-derived indices, such as Triglyceride glucose-body mass index (TyG-BMI) or Triglyceride glucose-waist circumference (TyG-WC), which may not comprehensively reflect central obesity and whose predictive value may vary across different populations, TyG-WHtR not only incorporates dual information on both insulin resistance and abdominal obesity but also exhibits higher sensitivity and specificity in predicting cardiovascular risk and metabolic disorders (20, 21). Therefore, this study selected TyG-WHtR as the primary indicator for metabolic syndrome.

Research on the relationship between the TyG-WHtR index and OAB is limited. This study aims to utilize the extensive dataset from the National Health and Nutrition Examination Survey (NHANES) to conduct a comprehensive analysis, determining the relationship between the TyG-WHtR index and the risk of developing OAB.

## Methods

### Study design and population

**Figure 1:**
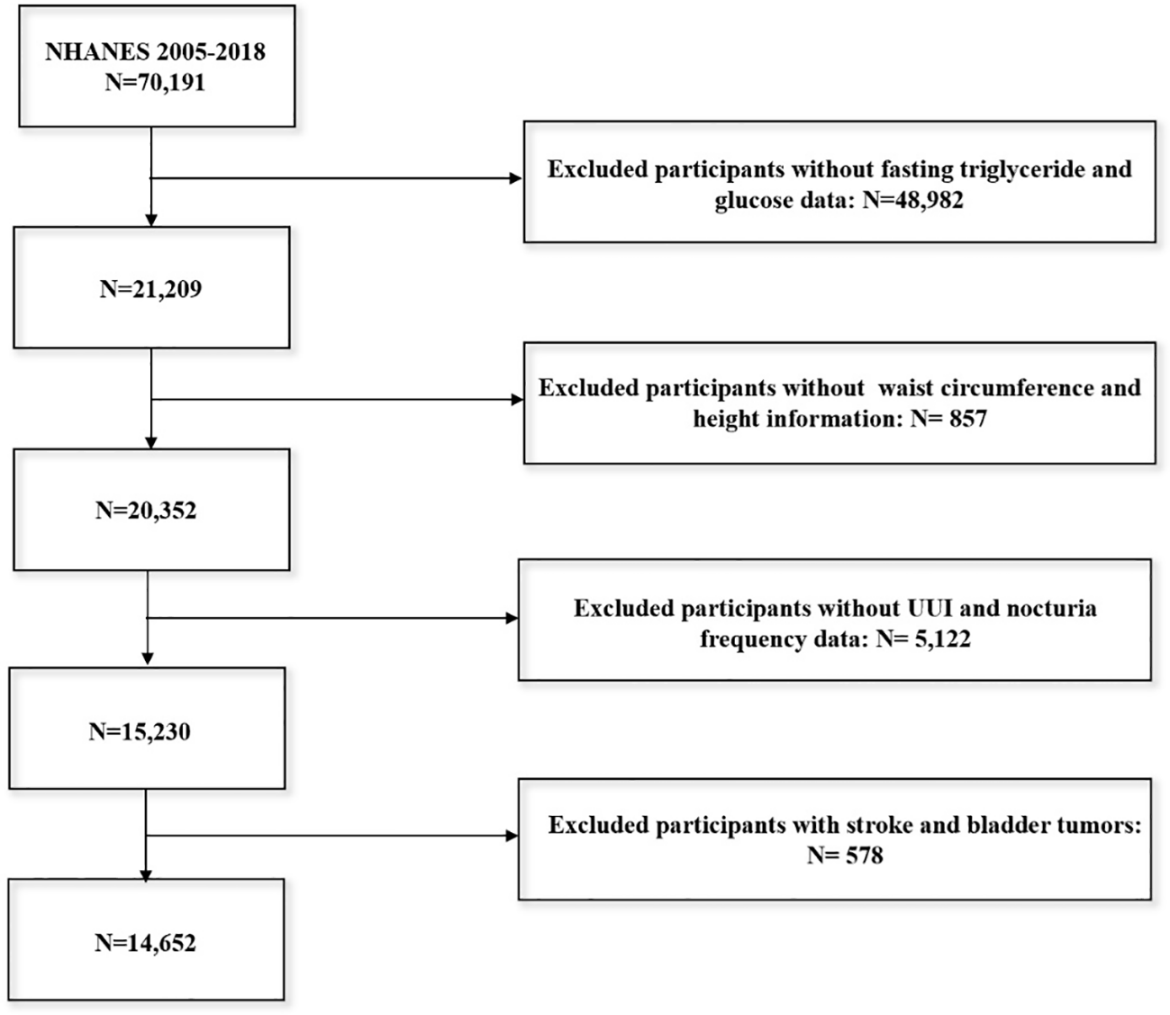
Screening conditions and process for the study population.

All data analyzed in this study were obtained from NHANES (https://www.cdc.gov/nchs/nhanes). NHANES is a nationally representative survey program that encompasses a wide range of health indicators, including clinical examinations, laboratory tests, and questionnaire-based data. It employs a complex sampling design to ensure that the sample is representative of the national population (22). Our analysis utilized data from seven cycles of NHANES, spanning from 2005 to 2018. During this period, 70,191 individuals participated in the survey. We excluded those with missing fasting triglyceride or glucose data (n=48,982), waist circumference or height information (n=857), and incomplete data on UUI and nocturia frequency (n=5,122). Additionally, participants with a history of stroke and bladder tumors (n=578) were excluded. After these exclusions, 14,652 participants were included in the final analysis. The specific flow of participant selection is detailed in **Figure 1**.

### TyG-WHtR measurement

The TyG-WHtR index is calculated using the formula: TyG-WHtR = TyG × WHtR (23), where TyG = ln [fasting triglycerides (mg/dl) × fasting glucose (mg/dl)/2] (24), and WHtR = waist circumference (cm)/height (cm). Participants had their fasting glucose and triglyceride levels measured after fasting for 9 hours. Height and waist circumference were measured by professionals at a mobile examination center.

### OAB diagnosis

The diagnosis of OAB was obtained following previous research (25). In simple terms, OAB is determined using the Overactive Bladder Symptom Score (OABSS). The OABSS includes scores for the severity of UUI and nocturia. An OABSS score ≥3 is indicative of OAB (26, 27). The NHANES assessed UUI and nocturia severity through a questionnaire conducted by trained professional researchers via face-to-face interviews. UUI was determined by the question “Urinated before reaching the toilet?” and its severity was assessed by asking “How frequently does this occur?” Nocturia severity was assessed with the question “How many times do you urinate at night?”.

### Demographic data, comorbidities, and other covariates

The covariates were selected according to the following protocol: demographic variables, including sex, age, race, and poverty income ratio (PIR), were included *a priori* as essential covariates. Additional covariates were identified and incorporated based on established risk factors for overactive bladder (OAB) as documented in the existing literature (20, 23, 28). The PIR is divided into three levels (“1,” “1-5,” and “5”) (29). Smoking history was defined as having smoked 100 or more cigarettes in a lifetime, while alcohol use was determined by whether the participant consumed 12 or more alcoholic drinks in a year. Medical history was based on self-reports of doctor-diagnosed diabetes, coronary heart disease, or cancer. Physical activity was categorized as vigorous or moderate intensity (yes/no). BMI was calculated as weight (kg) divided by height (m²) and classified into three categories: under 25 (normal weight), 25-29.9 (overweight), and 30 or above (obese). Laboratory indicators included albumin, alanine aminotransferase (ALT), aspartate aminotransferase (AST), total cholesterol, and glycohemoglobin. Kidney function was assessed using creatinine clearance rates calculated with the CKD-EPI Creatinine Equation (2021) (30).

### Statistical analyses

Statistical analyses were conducted according to the NHANES analysis guidelines and recommended weighting. The Kolmogorov-Smirnov test was used to assess the normality of the data. Continuous variables that were not normally distributed were presented as median ± interquartile range, and comparisons between groups were made using the Mann-Whitney U test. Categorical variables were presented as frequencies and percentages, with comparisons between groups made using the chi-square test.

Weighted multivariable logistic regression analysis was used to investigate the relationship between TyG-WHtR and OAB. Three models were constructed: Model 1 was unadjusted, Model 2 was adjusted for gender, age, and race, and Model 3 was adjusted for all variables. The TyG-WHtR was also divided into quartiles, and a weighted multivariable logistic regression analysis and trend test were performed. Prior to finalizing the model, we assessed multicollinearity by calculating variance inflation factors (VIFs) for all candidate covariates, with a predetermined threshold of VIF < 5 indicating acceptable levels of collinearity. To address missing covariate data, the multiple imputation (MI) method was applied, and sensitivity analyses were conducted to reduce potential bias. Subgroup analyses and interaction tests were further performed to investigate the influence of other risk factors on the association between TyG-WHtR and OAB risk. The generalized additive model (GAM) was used to calculate predicted probabilities, and smooth curve fitting and threshold effect analysis were used to examine the nonlinear relationship between TyG-WHtR and OAB risk. Additionally, receiver operating characteristic (ROC) curve analysis was performed to quantify the value of the TyG-WHtR index in predicting OAB occurrence, using the area under the ROC curve (AUC). The optimal cut-off value was determined using the Youden Index (sensitivity + specificity − 1).

All statistical analyses were conducted using R software (version 4.0.0), EmpowerStats (version 4.0), and SPSS (version 25.0), with a significance level set at P<0.05.

## Results

### Baseline characteristics

**Table 1** presents the baseline characteristics of the study population. A total of 14,652 adults aged 20 years and older were included in the study. After applying sampling weights, the estimated population size was 197,598,146.7, of which 97,332,840.89 (49.26%) were male, and 85,541,488.42 (43.29%) were aged 50 years or older. Among the 14,652 participants, 3,399 were diagnosed with OAB, corresponding to a weighted estimate of 37,872,284.55 individuals. The median TyG-WHtR for the overall population was 4.98, while the median TyG-WHtR for OAB patients was 5.44, which was higher than the median TyG-WHtR for non-OAB participants (4.88). The proportion of males in the OAB group was lower than in the non-OAB group (31.89% vs. 53.38%). The OAB group also had a higher proportion of participants aged 50 years and older, females, individuals with obesity, and those with lower physical activity levels. Additionally, the OAB group had higher levels of total cholesterol and glycohemoglobin, as well as higher rates of cancer, coronary heart disease, hypertension, and diabetes compared to the non-OAB group.

**Table 1:** Weighted baseline characteristics of the study population.

Variables	Total (n = 14,652)	Without OAB (n = 11,253)	With OAB (n = 3,399)	*P*
Weight N	Weight N =197598146.7	Weight N =159725862.1	Weight N =37872284.55	
TyG-WHtR, mean (Q_1_ , Q_3_ )	4.98 (4.29, 5.71)	4.88 (4.21, 5.58)	5.44 (4.73, 6.21)	<0.001
Gender, n (%)				<0.001
Male	97332840.89 (49.26)	85253809.11 (53.38)	12079031.78 (31.89)	
Female	100265305.80 (50.74)	74472053.03 (46.62)	25793252.77 (68.11)	
Age, n (%)				<0.001
<50	112056658.27 (56.71)	99875776.32 (62.53)	12180881.95 (32.16)	
≥50	85541488.42 (43.29)	59850085.82 (37.47)	25691402.59 (67.84)	
Race/Ethnicity, n (%)				<0.001
Mexican American	16980801.46 (8.59)	14091680.97 (8.82)	2889120.49 (7.63)	
Other Hispanic	11068078.99 (5.60)	9161028.10 (5.74)	1907050.89 (5.04)	
Non-Hispanic White	134243951.36 (67.94)	108317742.52 (67.81)	25926208.84 (68.46)	
Non-Hispanic Black	21140362.31 (10.70)	15917807.68 (9.97)	5222554.63 (13.79)	
Other race/multiracial	14164952.57 (7.17)	12237602.87 (7.66)	1927349.70 (5.09)	
Education level, n (%)				<0.001
Less Than 9th Grade	10028845.65 (5.08)	7184351.97 (4.50)	2844493.68 (7.51)	
9-11th Grade	20563548.77 (10.41)	15377279.87 (9.63)	5186268.89 (13.70)	
High School Grad/GED or Equivalent	45992610.76 (23.28)	36274719.82 (22.72)	9717890.94 (25.67)	
Some College or AA degree	61804829.15 (31.29)	50333275.01 (31.52)	11471554.14 (30.30)	
College Graduate or above	59162331.38 (29.95)	50523380.81 (31.64)	8638950.57 (22.82)	
Poverty ratio, n (%)				<0.001
<1	24970339.39 (13.46)	18853030.02 (12.54)	6117309.36 (17.38)	
1-5	112806082.37 (60.80)	90865922.59 (60.44)	21940159.78 (62.33)	
≥5	47768728.72 (25.75)	40623628.42 (27.02)	7145100.31 (20.30)	
BMI, kg/m^2^, n (%)				<0.001
Normal weight	59437517.95 (30.11)	51464495.45 (32.25)	7973022.50 (21.09)	
Overweight	65300327.48 (33.08)	54075692.36 (33.89)	11224635.12 (29.69)	
Obese	72659649.18 (36.81)	54044950.72 (33.87)	18614698.46 (49.23)	
Vigorous activity, n (%)				<0.001
Yes	48980300.39 (24.79)	41898959.19 (26.23)	7081341.20 (18.70)	
No	148600519.59 (75.21)	117812607.63 (73.77)	30787911.97 (81.30)	
Moderate activity, n (%)				<0.001
Yes	61151987.66 (30.95)	54398369.31 (34.06)	6753618.35 (17.83)	
No	136446159.02 (69.05)	105327492.83 (65.94)	31118666.19 (82.17)	
Cancer, n (%)				<0.001
Yes	17823127.04 (9.03)	11204275.07 (7.02)	6618851.97 (17.48)	
No	179622031.59 (90.97)	148379612.70 (92.98)	31242418.89 (82.52)	
Coronary heart disease, n (%)				<0.001
Yes	6104046.56 (3.10)	3893483.32 (2.44)	2210563.24 (5.87)	
No	190990093.10 (96.90)	155538653.26 (97.56)	35451439.84 (94.13)	
Hypertension, n (%)				<0.001
Yes	61894708.29 (31.36)	42968538.71 (26.94)	18926169.58 (49.99)	
No	135476384.50 (68.64)	116542176.24 (73.06)	18934208.26 (50.01)	
Diabetes, n (%)				<0.001
Yes	17249330.27 (8.73)	10399864.29 (6.51)	6849465.98 (18.10)	
No	176030941.67 (89.14)	146528529.11 (91.79)	29502412.57 (77.96)	
Borderline	4200690.67 (2.13)	2708228.54 (1.70)	1492462.13 (3.94)	
Smoke status, n (%)				0.002
Yes	88909303.84 (45.01)	70423060.04 (44.10)	18486243.80 (48.86)	
No	108607134.06 (54.99)	89258951.13 (55.90)	19348182.93 (51.14)	
Alcohol use, n (%)				<0.001
Yes	133208487.17 (68.12)	110790669.65 (70.01)	22417817.52 (60.11)	
No	62328442.74 (31.88)	47450047.70 (29.99)	14878395.04 (39.89)	
Total cholesterol, mg/dl, mean (Q_1_ , Q_3_ )	190.00 (164.00, 217.69)	189.00 (164.00, 217.00)	192.00 (166.00, 221.00)	0.031
Glycohemoglobin, %, mean (Q_1_ , Q_3_ )	5.40 (5.20, 5.70)	5.40 (5.10, 5.70)	5.60 (5.30, 6.00)	<0.001
Albumin, g/l, mean (Q_1_ , Q_3_ )	43.00 (40.00, 45.00)	43.00 (41.00, 45.00)	41.00 (39.00, 43.00)	<0.001
ALT, U/L, mean (Q_1_ , Q_3_ )	21.00 (16.00, 29.00)	21.00 (16.00, 29.00)	20.00 (15.00, 27.00)	<0.001
AST, U/L, mean (Q_1_ , Q_3_ )	22.00 (19.00, 27.00)	22.00 (19.00, 27.00)	22.00 (18.00, 27.00)	0.02
eGFR, mL/min/1.73m², men (Q_1_ , Q_3_ )	95.59 (80.71, 109.29)	97.38 (83.07, 110.83)	87.15 (70.99, 101.28)	<0.001

### Associations between the TyG-WHtR index and OAB

Weighted logistic regression analysis was conducted to explore the association between the TyG-WHtR index and OAB. As shown in **Table 2**, in the unadjusted model (Model 1), TyG-WHtR was significantly positively associated with OAB (OR=1.646; 95% CI: 1.562, 1.735; *P*<0.001). This positive association remained significant after adjusting for age, gender, and race/ethnicity (Model 2) (OR=1.518; 95% CI: 1.434, 1.608; *P*<0.001). Even after adjusting for all covariates (Model 3), the positive association between the TyG-WHtR index and OAB remained significant, with each one-unit increase in the TyG-WHtR index associated with a 1.310-fold increased risk of OAB (95% CI: 1.157, 1.484; *P*<0.001).

**Table 2:** Associations between the TyG-WHtR index and OAB.

	Model 1		Model 2		Model 3	
	OR (95% CI)	*P*	OR (95% CI)	*P*	OR (95% CI)	*P*
Tyg-WHtR	1.646 (1.562, 1.735)	<0.001	1.518 (1.434, 1.608)	<0.001	1.310 (1.157, 1.484)	<0.001
Categories						
Quartile 1	Reference	/	Reference	/	Reference	/
Quartile 2	1.767 (1.49, 2.095)	<0.001	1.512 (1.256, 1.822)	<0.001	1.411(1.121, 1.776)	0.004
Quartile 3	2.366 (1.992, 2.810)	<0.001	1.988 (1.670, 2.367)	<0.001	1.611(1.206, 2.153)	0.002
Quartile 4	4.260 (3.670, 4.944)	<0.001	3.195 (2.723, 3.750)	<0.001	2.034(1.419, 2.915)	<0.001
P for trend	/	<0.001	/	<0.001	/	<0.001

Model 1: unadjusted; Model 2: adjusted for gender, age, and race/ethnicity; Model 3: additional adjustments for education level, BMI, total cholesterol, vigorous activity, moderate activity, eGFR, hypertension, diabetes, coronary heart disease, cancer, ALT, AST, albumin, glycohemoglobin, alcohol, and smoke. OR: odds ratio; 95% Cl: 95% confidence interval.

Subsequently, the TyG-WHtR was divided into four quartiles, with Quartile 1 used as the reference group. After adjusting for all confounding factors, the odds ratios (ORs) with corresponding confidence intervals (CIs) indicated significant positive associations for Quartiles 2, 3, and 4 compared to Quartile 1. Specifically, compared to Quartile 1, the ORs for Quartiles 2, 3, and 4 were 1.411 (95% CI: 1.121, 1.776; *P*=0.004), 1.611 (95% CI: 1.206, 2.153; *P*=0.002), and 2.034 (95% CI: 1.491, 2.915; *P*<0.001), respectively. Additionally, the trend test (*P* for trend<0.01) indicated that the risk of OAB increased as the TyG-WHtR quartile increased.

### Sensitivity analysis

To evaluate the potential influence of missing data, we performed a sensitivity analysis using multiple imputation for missing values. Subsequent multivariate logistic regression analysis yielded results consistent with the original unimputed analysis (**Supplementary Data Table S1**), confirming the robustness of our findings.

### Subgroup analysis

To investigate whether the relationship between the TyG-WHtR index and OAB is influenced by potential confounding factors or effect modifiers, we conducted subgroup analyses and interaction tests based on gender, age, ethnicity, BMI, lifestyle factors (such as smoking, alcohol consumption, and physical activity), and medical history ((including hypertension and diabetes). As shown in **Table 3**, after adjusting for multiple confounding factors, the association between the TyG-WHtR index and OAB risk was stronger in women compared to men. When grouped by age, the association between the TyG-WHtR index and OAB risk gradually weakened with increasing age. These two subgroup interactions were statistically significant (interaction *P* values <0.05). However, when the subgroup analysis was stratified by BMI, lifestyle factors, hypertension, and diabetes, a positive association between the TyG-WHtR index and OAB risk was observed across all subgroups, with no significant interaction detected (interaction *P* values > 0.05). Among different race/ethnicity groups, the relationship between TyG-WHtR and OAB was most pronounced in Non-Hispanic Whites (OR=1.397, *P* < 0.001), while relatively weaker in other groups, with no significant interaction observed (*P* for interaction = 0.661). Overall, the relationship between the TyG-WHtR index and OAB risk showed variations across gender, age, and certain lifestyle factors, being particularly significant in women and individuals under 50 years old.

**Table 3:** Subgroup analysis for the associations between the TyG-WHtR index and OAB.

Variables	OR (95% CI)	*P*	*P* for interaction
**Overall**	1.310 (1.157, 1.484)	<0.001	
Gender			0.011
Male	1.260 (1.025, 1.548)	0.029	
Female	1.323 (1.138, 1.538)	<0.001	
Age (years)			<0.001
<50	1.426 (1.180, 1.724)	<0.001	
≥50	1.148 (0.992, 1.329)	0.064	
BMI			0.121
Normal weight	1.542 (1.176, 2.022)	0.002	
Overweight	1.371 (1.009, 1.862)	0.044	
Obese	1.251 (1.099, 1.423)	0.001	
Vigorous activity			0.109
Yes	1.480 (1.148, 1.910)	0.003	
No	1.278 (1.121, 1.456)	<0.001	
Moderate activity			0.628
Yes	1.508 (1.153, 1.971)	0.003	
No	1.276 (1.126, 1.447)	<0.001	
Hypertension			0.233
Yes	1.282 (1.099, 1.496)	0.002	
No	1.348 (1.164, 1.560)	<0.001	
Diabetes			0.148
Yes	1.282 (1.016, 1.616)	0.036	
No	1.348 (1.168, 1.556)	<0.001	
Borderline	1.357 (0.804, 2.290)	0.237	
Race/Ethnicity			0.661
Mexican American	1.143 (0.888, 1.471)	0.294	
Other Hispanic	1.115 (0.822, 1.511)	0.478	
Non-Hispanic White	1.397 (1.176, 1.66)	<0.001	
Non-Hispanic Black	1.171 (0.987, 1.389)	0.070	
Other race/multiracial	1.544 (0.998, 2.390)	0.051	
Smoke status			0.928
Yes	1.330 (1.145, 1.546)	<0.001	
No	1.328 (1.093, 1.615)	0.005	
Alcohol use			0.940
Yes	1.275 (1.095, 1.485)	0.002	
No	1.360 (1.113, 1.663)	0.003	

### Nonlinear association between the TyG-WHtR index and OAB

**Figure 2:**
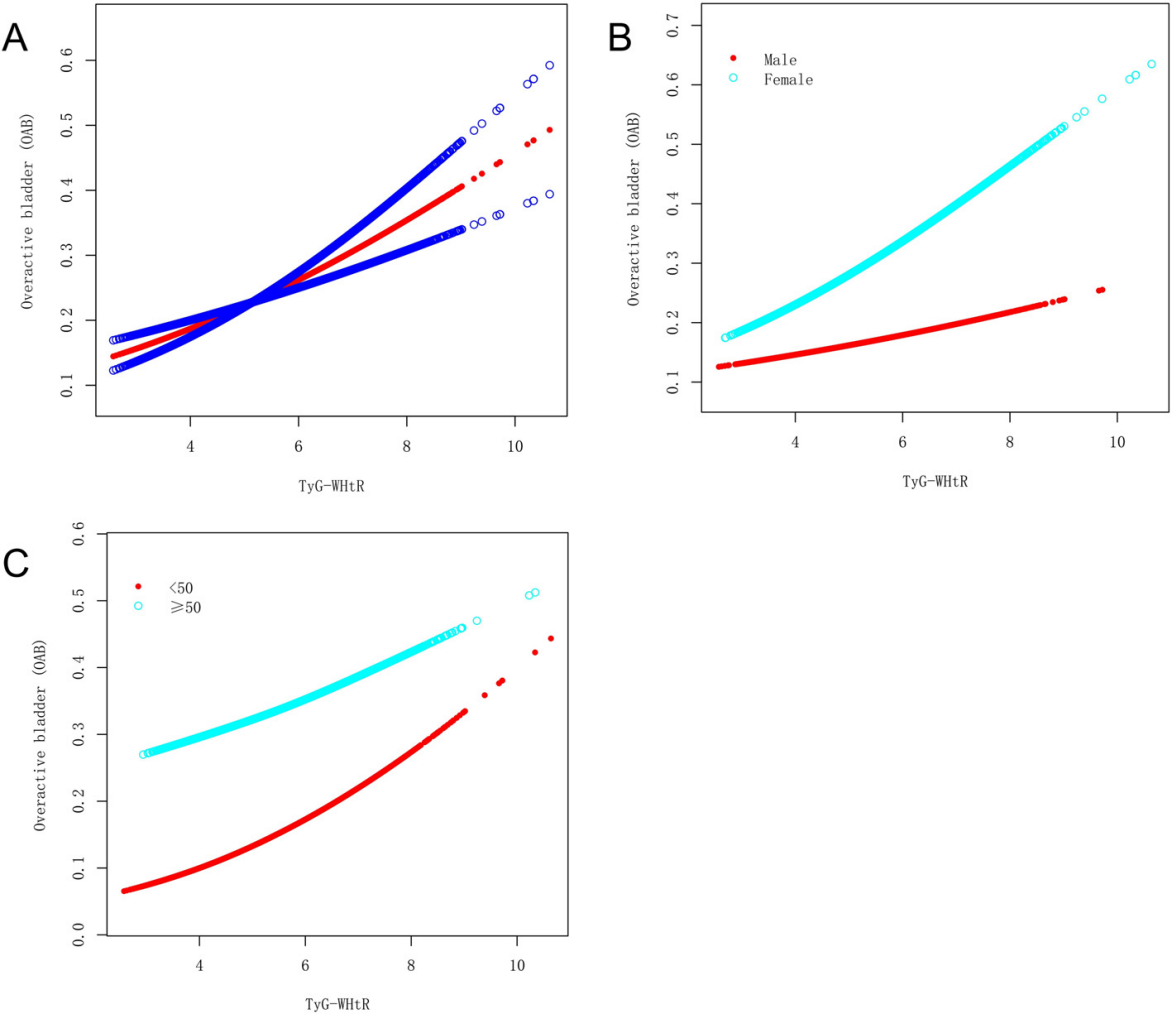
The association between the TyG-WHtR index and OAB. **(A)** The solid red line represents the smooth curve fit between variables. Blue bands represent the 95% confidence bands derived from the fit. **(B)** Stratified by gender. **(C)** Stratified by age.

A nonlinear relationship between the TyG-WHtR index and OAB was explored using a smooth curve fitting and threshold effect analysis, with the results shown in **Figure 2**, **Table 4**. A two-piecewise linear regression model identified a turning point at 3.579. When the TyG-WHtR index exceeded 3.579, a significant positive association between TyG-WHtR and OAB was observed (OR=1.228; 95% CI: 1.141, 1.322; *P*<0.001). Furthermore, we found a clear inflection point and saturation effect for the TyG-WHtR index in women and individuals over 50 years of age, with turning points at 3.568 and 5.583, respectively.

**Table 4:** Threshold effect analysis of TyG-WHtR index and OAB.

OAB	Adjusted OR (95% CI) *P* value
TyG-WHtR index	
Inflection point	3.579
TyG-WHtR <Inflection point	2.163 (0.668, 7.009) 0.1983
TyG-WHtR >Inflection point	1.228 (1.141, 1.322) <0.001
Log-likelihood ratio	0.334
Gender	
Male	
Inflection point	3.850
TyG-WHtR index<Inflection point	0.836 (0.371, 1.883) 0.6654
TyG-WHtR index>Inflection point	1.175 (1.032, 1.337) 0.0147
Log-likelihood ratio	0.430
Female	
Inflection point	3.568
TyG-WHtR index<Inflection point	11.441 (1.263, 103.639) 0.0302
TyG-WHtR index>Inflection point	1.257 (1.147, 1.378) <0.0001
Log-likelihood ratio	0.028
Age (years)	
Below50	
Inflection point	6.891
TyG-WHtR index<Inflection point	1.296 (1.118, 1.502) 0.0006
TyG-WHtR index>Inflection point	0.960 (0.690, 1.334) 0.8073
Log-likelihood ratio	0.134
Over50	
Inflection point	5.583
TyG-WHtR index<Inflection point	1.017 (0.865, 1.196) 0.8404
TyG-WHtR index>Inflection point	1.263 (1.113, 1.432) 0.0003
Log-likelihood ratio	0.048

### ROC curve evaluation of the TyG-WHtR index in predicting OAB events

**Figure 3:**
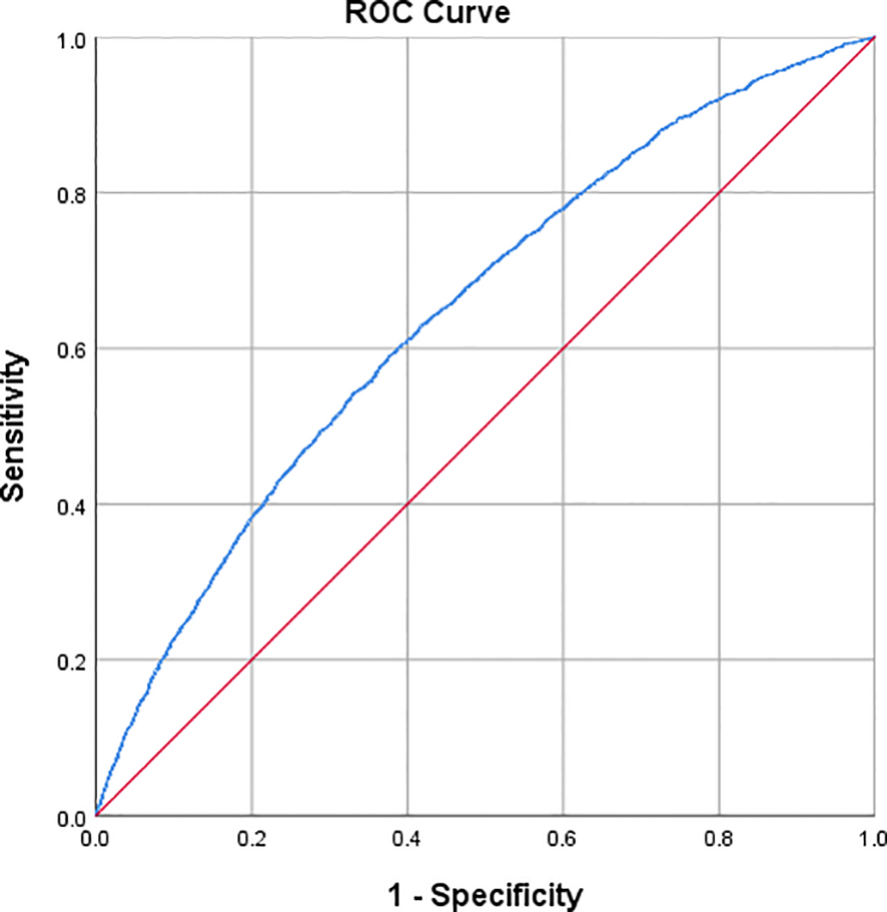
Receiver operating characteristic curves for baseline TyG-WHtR predicting OAB.

The predictive accuracy, sensitivity, and specificity of the TyG-WHtR index for OAB events were evaluated using ROC curve analysis. As shown in **Figure 3**, the TyG-WHtR index demonstrated a good ability to predict OAB events (AUC=0.647; 95% CI: 0.636, 0.657). Based on the Youden Index, the optimal cut-off value for the TyG-WHtR index in predicting OAB events was 5.261, with a sensitivity of 0.592 and a specificity of 0.622.

## Discussion

In this study, we investigated the association between the TyG-WHtR index and the occurrence of OAB in a large cohort of American adults. We found a significant positive association between the two, and this association remained robust even after adjusting for various potential confounders. Sensitivity analyses were conducted to validate the robustness of the findings. Subgroup and interaction analyses revealed that the impact of the TyG-WHtR index on OAB may be influenced by gender and age, emphasizing the importance of considering these factors when assessing the relationship between the TyG-WHtR index and OAB. Furthermore, curve fitting analysis and threshold effect analysis demonstrated a highly significant positive relationship between the TyG-WHtR index and OAB. Additionally, the ROC analysis showed that the TyG-WHtR index has good predictive ability for OAB. Therefore, the TyG-WHtR index may serve as a novel risk predictor for OAB.

The TyG-WHtR index combines triglycerides, fasting glucose, and waist-to-height ratio, and it is closely associated with insulin resistance, hyperuricemia, diabetes, non-alcoholic fatty liver disease, and cardiovascular diseases (23, 28, 31–34). This study reveals a significant positive association between the TyG-WHtR index and OAB, suggesting a potential biological connection between metabolic syndrome markers and bladder dysfunction. Elevated TyG-WHtR levels may increase OAB risk through several mechanisms. First, insulin resistance and obesity can trigger systemic inflammation (23). Inflammatory mediators like tumor necrosis factor-α (TNF-α) and interleukin-6 (IL-6) may disrupt bladder neural and muscular sensitivity, leading to functional abnormalities in the bladder wall. This can cause fibrosis or stiffness in the bladder’s smooth muscle, reducing its adaptability to capacity changes and triggering overactive symptoms (7, 12, 35). Additionally, insulin resistance and obesity can impair endothelial function, causing arteriosclerosis and microcirculatory disturbances (36), which compromise blood and nutrient supply to the bladder, increasing sensitivity and contraction frequency (7, 8). Hormonal imbalances due to these conditions may also disrupt the sympathetic-parasympathetic nervous system balance, affecting bladder contraction and voiding (37). Furthermore, individuals with high TyG-WHtR levels often exhibit autonomic nervous system dysfunction, which can impair bladder control mechanisms (12, 38). Dysregulation of the autonomic nervous system may lead to detrusor muscle overactivity and urethral sphincter dysfunction, exacerbating OAB symptoms (39). Unhealthy lifestyle factors associated with high TyG-WHtR, such as high-calorie or irritant-rich diets, may also contribute to bladder dysfunction. For instance, excessive sugar or fat intake can directly affect bladder sensitivity and contraction (40, 41). In summary, the TyG-WHtR index’s association with OAB likely involves multiple pathways, including insulin resistance, adipose tissue distribution, inflammatory responses, and neuroendocrine dysregulation, collectively impairing bladder function and promoting OAB development.

Exploring subgroup analysis and interactions is crucial in clinical research to better understand the actual relationship between independent and dependent variables (42). In this study, we conducted subgroup analysis and interaction tests using gender, age (grouped by 50 years, the median age of the overall population), BMI, race, lifestyle factors (smoking, alcohol consumption, and physical activity), hypertension, and diabetes as stratifying variables. We found that the TyG-WHtR index had a stronger association with the risk of OAB in women and those under 50 years of age. This may be due to more pronounced hormonal levels, lifestyle factors such as dietary habits, stress levels, insulin resistance, and obesity in these two groups, which in turn affect bladder sensitivity and detrusor muscle activity, thereby increasing the risk of OAB (7, 37, 40, 41). While these findings hold significant implications for the U.S. population, it is important to acknowledge that the generalizability of the results to a global context requires careful consideration. Cultural, lifestyle, dietary, and healthcare system differences across countries and regions may influence the relationship between the TyG-WHtR index and OAB. Therefore, although this study provides valuable insights into the association between the TyG-WHtR index and OAB, future research should validate these findings in diverse cultural and lifestyle settings to ensure their global applicability. In summary, subgroup analyses indicate that the positive association between the TyG-WHtR index and OAB risk remains consistent across most populations, particularly among women and individuals under 50 years of age. These results suggest that the TyG-WHtR index could serve as a valuable indicator for predicting OAB risk, with promising potential for application in OAB risk screening. However, its broader applicability warrants further validation across different cultural and lifestyle contexts.

This study has several strengths. First, to our knowledge, this is the first study to evaluate the risk of OAB occurrence associated with the TyG-WHtR index. Clinically, the TyG-WHtR index is a convenient, economical, and easily obtainable indicator. Second, this study is based on a large, diverse cohort with a wide age range, providing a sufficient sample size to ensure the reliability and stability of the results. However, this study also has some limitations. First, as a retrospective study, it cannot establish causality. Second, despite considering many factors in our analysis, some potential confounders may not have been assessed due to the limitations of NHANES data. Third, symptoms of nocturia and urinary incontinence were collected through questionnaires, which may introduce recall bias. Additionally, factors such as genetic background, dietary patterns, and lifestyle differences may influence the generalizability of these findings.

## Conclusion

The results of this study indicate a significant positive association between the TyG-WHtR index levels and the risk of OAB occurrence. The TyG-WHtR index could potentially serve as a novel predictive marker for OAB risk. Future research should include longitudinal and interventional studies to further validate these findings, explore causal relationships, and elucidate underlying pathological mechanisms. Additionally, evaluating the global applicability of these results across diverse cultural and lifestyle contexts, as well as developing multifactorial predictive models, could enhance early identification and risk assessment capabilities for OAB.

## Data Availability

The original contributions presented in the study are included in the article/**Supplementary Material**. Further inquiries can be directed to the corresponding author.

## Glossary

TyG-WHtR: Triglyceride glucose-waist height ratio
OAB: Overactive bladder
NHANES: National Health and Nutrition Examination Survey
ROC: Receiver Operating Characteristic
AUC: area under the ROC curve
UUI: urgency urinary incontinence
IR: insulin resistance
TyG: triglyceride-glucose
HOMA-IR: homeostasis model assessment of insulin resistance
TyG-WC: Triglyceride glucose-waist circumference
TyG-BMI: Triglyceride glucose-body mass index
WHtR: waist-to-height ratio
BMI: body mass index
SES: socioeconomic status
NCHS: National Center for Health Statistics
ERB: Ethics Review Board
OABSS: Overactive Bladder Symptom Score
PIR: poverty income ratio
ALT: alanine aminotransferase
AST: aspartate aminotransferase
MI: multiple imputation
VIFs: Variance inflation factors
GAM: generalized additive model
ORs: odds ratios
CIs: confidence intervals
TNF-α: tumor necrosis factor-α
IL-6: interleukin-6
