# Supplementary material for: Association between triglyceride glucose-waist height ratio index and overactive bladder: based on NHANES 2005-2018
Source: Front Endocrinol (Lausanne). 2025 Apr 15;16:1541218. doi: 10.3389/fendo.2025.1541218 (PMC12037367; doi:10.3389/fendo.2025.1541218)
Supplement: Supplementary file 1 [file Table1.docx]

**Table S1.** Sensitivity Analysis of the TyG-WHtR index and OAB.

|  | | Model 1 | | | Model 2 | | Model 3 | |
| --- | --- | --- | --- | --- | --- | --- | --- | --- |
|  | OR (95% CI) | | *P* | OR (95% CI) | | *P* | OR (95% CI) | *P* |
| TyG-WHtR | 1.646 (1.562, 1.735) | | <0.001 | 1.518 (1.434, 1.608) | | <0.001 | 1.297 (1.153, 1.460) | <0.001 |
| Categories |  | |  |  | |  |  |  |
| Quartile 1 | Reference | | / | Reference | | / | Reference | / |
| Quartile 2 | 1.767 (1.49, 2.095) | | <0.001 | 1.512 (1.256, 1.822) | | <0.001 | 1.301 (1.048,1.617) | 0.018 |
| Quartile 3 | 2.366 (1.992, 2.810) | | <0.001 | 1.988 (1.670, 2.367) | | <0.001 | 1.520 (1.156,1.998) | 0.003 |
| Quartile 4 | 4.260 (3.670, 4.944) | | <0.001 | 3.195 (2.723, 3.750) | | <0.001 | 1.916 (1.361,2.696) | <0.001 |
| *P* for trend | / | | <0.001 | / | | <0.001 | / | <0.001 |

Model 1: unadjusted; Model 2: adjusted for gender, age, and race/ethnicity; Model 3: additional adjustments for education level, BMI, total cholesterol, vigorous activity, moderate activity, eGFR, hypertension, diabetes, coronary heart disease, cancer, ALT, AST, albumin, glycohemoglobin, alcohol, and smoke. OR: odds ratio; 95% Cl: 95% confidence interval.
